# Motivated leadership and mutual learning: a case study of promoting sustainable food consumption in an early childhood education centre in Aotearoa New Zealand

**DOI:** 10.1080/03036758.2024.2410010

**Published:** 2024-10-09

**Authors:** Nicole Poindexter, Kathryn Bradbury, Janine Wiles

**Affiliations:** School of Population Health, Faculty of Medical and Health Sciences, The University of Auckland, Auckland, New Zealand

**Keywords:** Plant-based, early childhood learning, community, sustainability, zero waste

## Abstract

Food system transformation is urgently needed; although dominant food and sustainability discourses tend to focus on powerful stakeholders like governments and industries. We sought to understand how localised, community-based initiatives promote food system change, and how this is experienced by the people they are meant to involve or benefit. We conducted a reflexive qualitative case study with a community-oriented early childhood centre in Aotearoa New Zealand that developed an increasingly sustainable programme. This included providing plant-based food, a garden-to-table and nature-oriented pedagogical focus, and well-embedded low waste practices. We engaged with the community of school leaders, staff, and whānau to explore their processes, their experiences of changes made, and their challenges. Our case study methodology involved semi-structured interviews, group discussions, document analysis, and a reflexive thematic analysis. We identified two main themes, including (1) the importance of leadership for initiating and sustaining change; (2) experiences of changes were linked to relationships and shared learning. These findings contribute to understanding the complexities involved in shifting to sustainable food practices at a local, community-focused level; these might benefit other communities or the stakeholders working to better support them.

## Introduction

The global food system is a significant contributor to greenhouse gas emissions (GHGs) and other pollution responsible for anthropogenic conditions (Springmann et al. 2016; Swinburn et al. 2019; Willett et al. 2019). Collated data from 1990 to 2015 (Crippa et al. 2021) show approximately one-third of global GHGs are tied to the food system, mostly from agriculture and its associated changes in land use, but also from supply chains, energy use, and waste practices. The deadly effects of higher GHGs include increased mortality from vector-borne diseases, heatstroke, and natural disasters (Kakalou and Tsiamis 2021; Chaves et al. 2024; Saatchi et al. 2024). With increased GHG emissions from meat and dairy production in particular, along with associated changes in global diets over the last few decades, these effects confound increased mortality from non-communicable diseases (NCDs), particularly cardiovascular disease (Springmann et al. 2016).

The EAT-Lancet Commission (Willett et al. 2019) reported that the health of people and the planet requires a ‘Great Food Transformation’ away from heavy consumption of meat and dairy to more plant-based diets, among other changes. Shifting to more sustainable, secure, and equitable food systems will require fundamental change, at all levels (Shukla et al. 2019; Swinburn et al. 2019; Willett et al. 2019). Challenging the inertia of systemic and institutional drivers of obesity, undernutrition, and climate change requires urgent solutions and effective actions. Swinburn et al. (2019, p. 289) see opportunities for local action:

People can leverage their individual agency better within their local school, grocery store, or workplace for small changes than they can in the education system, the food system, or businesses at large for big changes. However, many small changes in communities can build into wider social change, especially if the local actions spread by creating virtuous cycles of mutual learning between communities.

Swinburn et al. (2019) emphasise the need to reorient systems locally and at larger scales. In this study, we focus on a community case study of an early childhood education centre to investigate how food sustainability efforts can be developed and sustained in a local setting, the impact and experiences of changes for community members, and how a community can manage the challenges they face.

### Literature review

Studies from diverse community contexts have explored sustainable food practices including shifts to greater plant-based food provision and consumption. A Portuguese-based study of school-meals interviewed multiple stakeholders to gain insights into how to increase plant-based eating in schools and more generally (Graça et al. 2022). They draw from the COM-B model for behaviour change (capacity, opportunity and motivation together lead to behaviour shifts) and suggest that beliefs about sustainability and animal welfare practices could be leveraged to build motivation for more plant-based consumption. Upskilling staff and improving meals on offer, as well as food-related literacy, could improve capacity for changes to be made. Structural supports such as public funding, dietary guidelines or suggestions, and education from outside of the school community offer opportunities. They also argued that changing social norms (e.g. that ‘proper meals’ are meat-centric) and active involvement of the wider school community were essential to support plant-based eating (Graça et al. 2022). Also emphasising the value of new learning, experiences, and ideas about food, Crary et al. (2022) studied 8–14-year-old children attending a farm-based camp in California. Campers were involved with harvesting, preparing, and cooking vegetables, and they found that children were more likely to choose vegetables that they prepared, expressing pride and ownership for their production efforts.

A mixed-methods study of a farmer-led, community-supported agriculture (CSA) movement in Hungary, where members subscribe for regular delivery of a box of produce, highlighted that relationships between farmers and members were central (Balázs et al. 2016). Farmers developed relationships with communities by teaching them about the seasonality of fruits and vegetables and making suggestions about how to prepare food. Members placed a high level of trust in the farmers, and felt a sense of community, valuing the shortened distance between production and consumption and the focus on cooperation and sustainability (Balázs et al. 2016).

The way in which food transitions are discussed and encouraged may also have an impact on the success of the implementation. Carvalho et al. (2022) found that University students were more supportive of strategies to encourage plant-based eating when these strategies were framed in terms of gain (i.e. promoting or increasing consumption of plant-based meals) rather than loss (i.e. reducing or curtailing meat-based meals). They also observed that impact for sustainable change was higher for more sensory-focused measures (e.g. making plant-based meals tastier and more appealing) than for behavioural constraints (serving only plant-based meals).

Spadafore (2020) explored how participation in a peer support group might lead to reduced meat consumption and related environmental actions. Employees from multiple organisations in an Ontario office building met with a researcher-facilitator in groups for a three-month participatory action research study. Support group participation did lead to reductions in meat consumption, however, once the group meetings ended, participants were more likely to return to practices and habits of the dominant food environment. If new learning and experiences are outside of the mainstream, shifting habits might be more effective if social support is meaningfully sustained.

Thus, promoting more sustainable food practices in schools and other communities extends beyond a focus on food provision and consumption to involving communities in food production and preparation. In addition, relationships are central to the community and how it interacts with food producers and any food transition, and the way a food transition is framed may influence a community’s receptiveness to that transition.

Previous studies have explored components of promoting sustainable food transitions in local communities. Understanding how communities make these shifts for themselves, and engage their membership in the process, could be helpful for supporting their agency as stakeholders in food system transformation. We were interested in the processes involved in transitioning to sustainable food practices, how the changes have been experienced, and how challenges are identified and managed. We therefore conducted a qualitative case study of an Early Childhood Centre (ECE) in Aotearoa New Zealand that transitioned to provision of plant-based and low waste food. The specific research questions were:

1. Why did the community transition to a sustainable food programme through plant-based and low-waste food?
2. What processes were involved in making the transition?
3. How did the community experience the changes and what challenges were faced?

We used a case study methodology to engage with the community of early childhood centre leaders, staff, and whānau to explore their experiences of their food transition.

## Methods

We conducted a qualitative case study (Merriam and Tisdell 2016), to develop an holistic, contextualised understanding of why and how this community transitioned to more sustainable, plant-based and nearly zero-waste practices. A case study allows for an in-depth description of multiple aspects of process and experience as well as for researcher interaction and engagement with an environment to understand complex aspects of a setting (Baum 2016; Merriam and Tisdell 2016). For this case study, we sought a community that had recently moved to becoming more sustainable. We reasoned that such a community would have engaged in the difficult conversations that larger society also must have to shift away from dominant ‘norms’ for food production, consumption, and waste.

An ECE on the outskirts of a regional city run by school leaders and staff committed to sustainability met our criteria as a community making changes to usual practices and policies to live and work more sustainably. At the time of the study, 45 families with children aged between 2 and 5 years old attended between 1 and 5 days a week (up to 30 children were present at any given time). The Centre employed 12 staff including teachers, a chef and two part-time aides.

We made contact to discuss a possible research project, visited the site, and developed the research questions collaboratively with the Head of School (HS), Head Teacher (HT) and two staff. They liked the idea of sharing their stories, and particularly wanted to explore any ‘ripple effect’ of the site’s sustainability practices for the wider community. We also discussed the first author’s positionality as Tauiwi [non-Māori] and an educator with personal and disciplinary interests in the food system. The research was approved by the University of Auckland Human Participants Ethics Committee.

Case study participants were the Centre staff and whānau [family groups] of the children attending the Centre, with the Centre (and its sustainability practices and their impacts) being the core unit of analysis (Merriam and Tisdell 2016, p. 39). The perspectives and experiences of participants were gathered through semi-structured interviews and group discussions, alongside observations over six weeks, and an analysis of a selection of Centre documentation (Bowen 2009). The latter included strategic plans, reviews and evaluations, communication with whānau and wider community, meeting agendas and minutes, notices of events, and menus.

The case study was introduced to participants in the Centre through a posted flyer and informal conversations between the first author and those who worked or brought children to the site. A summary of the research objectives and questions was provided for potential participants that outlined how data would be collected and stored. To avoid any sense of coercion, considering the interest in the research of the site owner, consent forms spelled out how participation in the research was voluntary and offered assurance that it would not impact employment or families’ relationships with the site; this was reiterated by the first author in conversations introducing the project. All those participating in interviews or group discussions gave written consent.

At the suggestion of the HS, the first author sat near the sign-in desk morning and afternoon over three weeks of data collection to meet whānau during drop-off or pick-up times, a kanohi ki te kanohi [face-to-face] strategy to introduce them to the research, provide contact information and consent forms, and engage whānau interest in participation. Some interviews took place at the site in a private space and other interviews were organised for a later time (either at the public library or, if it was easier for the family, at their home). Given the frequent use of a local bulk food shop by Centre staff and whānau, a $10 voucher for that shop was provided as a small thank you for participating in the research.

Nineteen parents participated in interviews, including three couples; in one interview, two children also participated. Interviews on site with whānau lasted between 15 and 30 min (as they were often on their way to or from work), three interviews were conducted with whānau at their home and were about 60–90 min each. Greeting potential participants in the morning also allowed for many informal chats with parents and staff, along with observations of the rapport between children and adults around food, casual conversations between families and the HS and the chef, and opportunities for sampling the food they produced. Audio memos created outside of site visits and interviews over the three weeks of data collection supported stronger recall and sense-making of findings from observations.

All 12 staff members were invited to participate in interviews through a conversation with the first author at a weekly staff meeting. Eleven participated in an individual interview, six also participated in one of two group interviews (led by the first author), and a former staff member who volunteered at the Centre also joined one group session. The 12th staff member did not want to be recorded, though articulated support of the research when on-site.

Individual interviews with staff lasted between 15 and 40 min with two longer (up to 90 min) interviews; the two staff group interviews were 60 and 90 min respectively. Interviews were recorded with written permission and transcribed by the first author. The research questions guided the topics discussed, with a focus on evidence of the process, experiences, and challenges faced.

Documents selected for analysis included strategic and annual plans and calendars (six documents); 26 review and evaluation documents; 15 newsletters, email and online posts to families including responses; 25 staff meeting agendas and minutes over three-and-a-half years; eight documents advertising Centre events related to sustainability such as workshops about low waste; and eight documents outlining menus and nutrition information. Weekly staff meeting minutes, for example, demonstrated how sustainable practices were systematised and how events and communication regarding changes happened.

### Analysis

Data for analysis included interview transcripts, documents, and field notes documenting interactions and observations on site as well as reflecting on interviews and documents. The focus of analysis was always on the processes for change, and experiences of these, particularly including challenges. Drawing on Braun and Clark’s (2021) reflexive thematic analysis (RTA) and with the research objectives in mind (Trainor and Bundon 2020), analysis moved through; (1) familiarisation, including descriptions and summaries of data (e.g. from individual and group interviews and transcripts of these, observations, and documents); (2) identifying recurring patterns, outliers and contradictions, and interpreting these by developing codes; (3) categorising codes into themes; (4) discussing, interrogating, and reviewing themes with the research team; (5) identifying relationships between themes and across types of data and (6) reporting on the research, including a summary for the Centre. We have anonymised the Centre and in presenting our findings we identify participants by their role in the Centre (as staff, leader or whānau).

## Findings

The Centre is a well-loved and well-maintained site, in an older house with mature fruit trees. The furniture is recycled, the food is made from inexpensive staple ingredients, and the toys are made of wood. Over a decade, the Centre moved towards integrating a sustainability programme for food production, consumption, and waste in the context of ECE operations and community life (Table 1).

**Table 1.** Summary of themes.

The importance of leadership for initiating and sustaining change *Leaders could ‘dream big’ and systematise action* *Leaders recognise opportunities and build capacity for others.*
2) Relationships and shared learning *Shared vision of the ‘whole ethos’* *Learning as an ongoing, experimental, and shared process* *Viewing challenges as external and addressing them together is helpful*

### Theme 1: the importance of leadership for initiating and sustaining change

Leadership for guiding the change processes at the Centre and a focus on clear values was vital, both from formal (HS and HT) and informal leaders. Their efforts assured that sustainability-oriented values were integrated into Centre systems and processes, and the community was introduced to these changes in meaningful ways.

#### Leaders could ‘dream big’ and systematise action

The HS recalled that when she set out to open the school she wanted to ‘dream big’. She thought about ways to make the school environmentally sustainable and educational, and healthy for children:

So we were going to have cows so we could milk, get our own milk and have chickens and grow all our own vegetables and … try and basically [be] off the grid, you know, our own solar power, any way we could be sustainable.

Cows on the property turned out to be impractical, but she found she could challenge herself with her imaginings of self-sufficiency and employ these values in her establishment (and for the families who would come there). The HS explained how easy it would be to establish an ECE ‘by the catalogue’; that piles of mailings come to ECEs to market what is ‘needed’ for children. This Centre’s leaders instead asked: ‘Do we need all this stuff?’

Feeding children, the garden-to-table approach for food, and teaching children to respect Papatūānuku or the Earth and work with each other and their natural environment were clear priorities. The HS explained how, for example:

meals have always been served here because food is such an important part of building relationships, health and wellbeing; and having the children prepare the food, gather the food has been important for us.

These values were also discussed by many staff. Teachers talked to children about where food comes from, and the children’s education includes time in the kitchen to participate in making and serving up their own food. With a buffet-style service area low to the ground, children were encouraged to sample everything at least once. The HS recalled taking advantage of ‘teachable moments’, like when the trees in the garden start dropping fruit, to discuss the benefits of eating what is seasonally available:

Children just sitting on the front porch, just eating buckets of feijoas, just eating, and the same with the plums. And it's nice because we know that they come into season when you need them the most, when you need to be building your vitamin C … [we] get the children to realise that.

Staff participants described how it was a gradual process. The zero-waste initiative began with an informal auditing of the rubbish bins at the site, count what kinds of products led to waste, and then try to make relevant shifts. The chef realised that they could purchase more from a local bulk food store, so menu-planning and food preparation became increasingly from scratch.

The HS frequently mentioned ‘passion’ and ‘commitment’ as critical qualities for making changes. They prioritised establishing a culture of reflection and collaboration, partly by committing to systematic documentation (e.g. staff meeting minutes and yearly calendars) to sustain the changes, ‘as it is easy to slide back into old habits’ (see Table 2). For example, the staff meeting agenda and minutes included a template for checking in on changes in the food system.

**Table 2.** Document analysis, examples of Centre strategies for collaboration and reflection on systemic change.

Process	Evidence
Decision-making as an inclusive process	Agenda templates collaboratively completed. Seeking whānau, marae feedback as weekly agenda item Yearly calendars and newsletters show events to include whānau and wider community in decisions i.e. ‘Community Planting Day,’ and ‘Marae Hui re: Vision’ Transition to plant-based menu noted one April: ‘Sharing plant-based diet menu with whānau’; the following July, a plant-based whānau Matariki celebration highlighted Communication with whānau with specific name for actioning (e.g. ‘X to check [website] comments posted by whānau’).
Regular/’standard’ ECE policies and procedures adapted to integrate sustainability values	‘‘Health and Safety Policies’ include health of the environment Template for self-evaluation: Centre food goals are incorporated i.e. ‘making food from scratch’ and ‘growing enough kai [food]’ with notes of current actions i.e. ‘seedlings – planting more’ Notes about the effectiveness of progress, e.g. ‘not enough [garden] beds, not enough produce’ Revisions to goals i.e. ‘we need to think through what we plant to match it [to] our menu’

Applying the goals they were passionate about as leaders into tangible systems and formalising these systems within the day-to-day and yearly running and environment of the school/small business meant these became guidelines for other staff members.

About 5 years after the site opened, moves to having a vegetarian and then vegan menu began. Initially, leaders challenged themselves to become vegetarian and then dairy-free as a Centre. They were also influenced and supported by a chef they hired, who was vegan and often used a vegan cookbook for planning meals for the children.

The 2016 annual Matariki school dinner with whānau (part of an overnight stay at the marae for the Matariki festival) was completely plant-based and provided for by the Centre staff, a change from the usual pot-luck style meal. It received positive feedback; multiple parents emailed or posted about the quality of the food and wanted recipes from the chef. The Centre leaders felt encouraged by feedback and lead a more official shift to an entirely plant-based menu at the site. They organised informational evenings beforehand, brought in a nutritionist and invited families. Community buy-in was valued though the HT also reflected that as owner, she could, and should, initiate integration of sustainability goals for the site.

Curriculum delivery and staff reflection processes show how structures were used to formalise values. Broader community participation in making and integrating changes was encouraged; however, we argue that it was leadership initiative < risk-taking, and capacity to institutionalise the processes that sustained them.

#### Leaders recognise opportunities and build capacity for others.

Those without formal leadership titles or responsibilities also had the capability, opportunities, and motivation to shift community sustainability practices. Often, they enabled other staff to experiment with more sustainable ways of doing things by sharing their experience and expertise. As a staff group participant reflected:

We also had a couple, when I started, a couple of vegan teachers here, and that really helped the process as well, they had experience to draw from, and they were passionate about it and wanted to push it quite hard as well. Yeah, definitely helped to get that going. (SG.1.1)

Having someone with the capability or ‘know-how’ for cooking with tofu or planning and cooking meat- and dairy-free meals promoted buy-in from others at the site. For example, the site chef offered opportunities for community members to try new foods. She focused on foods that families would not consider ‘weird stuff’ so they would be open to trying new things. Using online communication also allowed staff and whānau members to share their knowledge and encourage experimentation. For example, staff described posting recipes or zero-waste ‘hacks’ on the website, such as how to upcycle old towels to make washcloths.

It was apparent that staff often shared the site’s food-related sustainability values and objectives. One staff member explained the philosophy had developed to be ‘about walking softly on the Earth’.

I think it has been a natural progression, really … . The way we treat each other, how we eat, what the environment looks like, social justice … . It was all a natural progression. And the idea about providing food from the beginning was important because we wanted to make sure that the tamariki [children], at least we knew when they were here, they were getting good, nutritious kai [food]. That way, if parents were busy when they went home and their child only had toast for tea, that was OK because they could count on the fact they had had a good kai. (SG1.2)

Staff often spoke passionately and openly about their own values, which were not the same as each other’s but generally reflected the values upon which the Centre was founded. For example, in a group interview, one staff member described a personal transition to a more plant-based diet but stated that they still occasionally went hunting. For them, the more direct connection between food production and consumption was reducing environmental damage. In the same group discussion, another talked about transitioning to vegetarianism after considering how animals suffered in meat production processes, while another identified as being more strictly vegan ‘for all of the reasons’.

### Theme 2: relationships and shared learning

Experiences of the transition to sustainability and changes thus made varied widely amongst the Centre community. Leadership and staff at the Centre linked this variation to relationships and opportunities for shared learning. It was also clear that various whānau and staff had arrived to the community at different stages of an ongoing process and thus had different experiences of the process and outcomes.

#### Shared appreciation of the ‘whole ethos’

The Centre’s kaupapa, it’s vision, ongoing work, and aspirations were regarded highly for what one parent referred to as ‘the whole ethos’, although people did have varied attitudes towards and experiences of specific aspects of it. When asked how the food programme influenced their decision to choose this Centre for their child:

[It was] more the outdoors stuff that I was more interested in, the big focus on outdoor play … it was icing on the cake that they were into sustainable practices … . We thought [the food] may help [my child] to eat more vegetables … definitely … and it's really implanted, caring for plants … yeah, it was the whole ethos, the food side was a part of it for us but not the whole thing. (W7)

Parents typically appreciated the sustainability messages being taught to their children and saw this as consistent with Centre practices and ‘nature pedagogy’. Another chose the Centre because ‘we do live relatively close but yeah the space and the nature-focus … [the] whole package, really.’ (W1) They liked that their child eats plant-based there, though they do not at home.

Attitudes about the transition to serving only plant-based food varied but were primarily positive. Several parents thought having plant-based food during the day was a healthy choice as their family either limited their own dairy and meat intake or valued the quality and nature of food served. One whānau felt their own diet (heavy in meat, dairy, and processed foods) was challenged when their child began at the Centre, but for another Mum, eating more plant-based and using less waste were also linked with her aspirations as Māori as it was better for the environment which is taonga [treasured]:

[Low waste] connects with us as Māori as our ancestors grew up in a time where they literally used everything from the land … . In the same awa [water body] they have an area where you wash, where you bathe and another area for your clothes … . Being [at the Centre] is going back to our own ways of living, going back to the whenua as we call it, of the land. (W5)

The family started replacing meat-based meals with vegan or vegetarian ones at home a few times a week. They found this difficult to sustain because of time constraints, but also observed it to be beneficial to their health.

For others, how much the practices at the Centre led to different practices at home is difficult to parse. Some families linked being more vegan in practice, or ‘vegan-curious,’ as one put it, to only eating sustainably sourced meat and making food from scratch. This was not from misunderstanding what it meant to be vegan but from prioritising the environment and less processed foods over strict adherence to only plant-based products:

My husband and I have always just eaten meat that we [hunt]; we shoot deer, but apart from what we’ve gathered, we try not to purchase meat. But we do eat meat and are not vegan ourselves, but I applaud anyone who is. We are always learning … . (W3)

Families who grew up hunting or on farms did not express tensions about having children at a vegan school; their ideas about living close to the land aligned enough with the Centre’s values.

Some families preferred the plant-based menu because they enjoyed that their kids were not getting processed meats (‘defrosted sausage rolls’ were often mentioned as a classic staple of the ECE environment). For some parents, meat was part of ‘a balanced diet’ though they did not mind the Centre only serving plant-based food to their kids when they were there. One parent noted their child only ate plant foods at the Centre and credited the chef’s skills.

Commentary around a negative reaction from ‘a vocal minority’ was present in both staff and whānau interviews. Specifically, two parents identified that there was concern from some other parents who felt the need to ‘supplement’ their children’s diets with more meat, eggs, and dairy in other parts of their day:

When they did decide to go fully plant-based, I was quite surprised, they got quite a lot of pushback from parents … I am not really sure with numbers as I was really happy with it … probably only a handful but the most vocal. (W8)

Others directly expressed that they could not understand some of the Centre’s practices; however, this did not change their mind about their child’s attendance there or their own participation with site activities. ‘The whole ethos’ was more important to them than one or two aspects they did not align with personally. For example, when the Centre became vegan, they still kept rescue hens on the property but thereafter donated the eggs, and this frustrated some parents who thought they should be part of the site’s menu. Discussion about this was widely and affectionately referred to as ‘Egg-Gate’, arguably demonstrating how different views could be engaged with in a way that did not appear to jeopardise the overall experience of the Centre or its practices.

When asked about the para kore [zero waste] efforts, there was a mixed response from ‘that’s an extension of what we do at home’ (W1) to ‘it’s difficult’ because of the expense, commute, and planning involved in buying food in bulk (W7). Some whānau also experienced stress and felt incapable of adapting to these practices. Two parents described sometimes unpacking packaged food into lunchboxes for ‘lunchbox day’ as they felt self-conscious for buying packaged food, although they did not suggest there was any shaming involved or particular attention called to the lunchboxes by Centre staff; nor was this observed on site.

When asked about the impact, some families stated there was no impact for them at home but, notably, also shared anecdotes about their child doing something they had learned at the Centre, such as planting seedlings at home. Others described how their children started to pick up litter when out in parks or other settings and talked with their families about waste.

#### Learning as an ongoing, experimental and shared process

The Centre emphasised partnership and inclusivity in embracing values of manaakitanga [hospitality, kindness, generosity, support] and kaitiakitanga [guardianship, stewardship], and the ongoing and shared learning about sustainability and sustainable practices. For staff participants, the impact of the ongoing transition to sustainable food was highlighted:

It was quite interesting watching the impact that it had, too, on the staff … it feels it is like a religion in a way, I feel like I want to tell people about it … . It came from that idea, that commitment, walking softly on the earth and what that looks like. (SG1.2)

This comment reflects the enthusiasm for the collaborative effort felt by the staff; ‘walking softly’ was a phrase used often and by multiple people in staff group discussions. One staff member described her own ‘journey’ further as to-ing and fro-ing with vegetarianism until it occurred to her that, ‘I can just make my own rules’; namely, to become more sustainable despite not fitting neatly into a category of ‘vegetarian’ or ‘vegan’. Another, frustrated with produce packaging, started growing more of their own: ‘I’ve turned my swimming pool at home, much to the disgust of the children, into a vegetable garden’ (SG1.4).

For staff who already identified as vegetarian, plant-based or vegan, moving to a vegetarian and then vegan menu at the site, going low waste, and growing the garden-to-table programme were still impactful, whether through learning about nutrition, low waste or how to grow vegetables:

The nutritional aspect for me specifically … . I’ve been vegan for ages. I like seeing what food to incorporate to get nutrition. (SG1.3)

Teaching children to grow seedlings was sometimes also a lesson for teachers:

No-one ever taught me how to grow vegetables … to learn to plant them and when the seasons are . … I think that’s so important for vegan, zero waste. So important. I’m learning along with the kids at the moment … . And it gives us connection. (SG1.3)

Teachers described ‘learning alongside’ or ‘with’ kids and families through a shared culture of experimentation and ‘let’s try to do this better’. This was echoed by parents: ‘[I like] that it’s understood by the children, so it’s not just [staff] efforts … the efforts are shared with the tamariki [children]’ (W3). Most ‘sustainable’ practices were part of day-to-day experiments which were documented before and after. Shifts were possible because of a sense of shared responsibility and effort; solutions found through dialogue and trial-and-error. There was an expectation of experimentation, rather than prescription; a sense that co-construction was worthwhile and fun.

The ‘journey’ was often described by both whānau and staff as a path with steps going back and forth with room for experimenting to become better kaitiakitanga, guardians and stewards of the environment. They often described how they felt personal responsibility for the collective work to be done. This shared learning seemed a well-developed part of the culture in and around the Centre, though specific practices were not necessarily experienced by all community members in the same way.

### Viewing challenges as external and addressing them together is helpful

All participants expressed motivation to be more sustainable but many also described how they must manage their efforts despite or around structural barriers. Collaborative efforts, social support, and explicitly encouraging experimentation and learning from mistakes helped them manage some challenges.

Staff members and whānau all identified aspects of cost, accessibility, affordability, and a lack of time as external barriers to eating more plant-based and low waste. As one parent described:

Um, price … . We get organics from [a local organic store], and it's expensive. And that’s something that we’re prepared to put our money toward but if you don’t have extra money … and it’s more taxing, more organisation - you’ve got to do your order on Sunday. (W1)

The same parent described the difficulty of sustaining this, saying their ‘fall over’ is ‘snacks’:

Yeah, snacks … . I actually work full-time, so making beautiful crackers that would get hoovered in one sitting just isn’t reality for me, so … . If I look at what’s in my shopping trolley, um, it's bread that’s packaged, bread in plastic and corn thins in plastic. (W1)

Some held in mind the open hours, distances, and pricing at different grocery vendors in town to weigh time and cost constraints against personal sustainable consumption goals, i.e. avoiding plastic in their shopping cart. If the inexpensive bulk food shop closed before the end of their work day, could they leave work early to get there? The decision fatigue of emotional labour involved weighed on parents. Sometimes, these challenges were internalised in disempowering ways. Parents often discussed feelings of shame, guilt, and a sense of ‘overwhelm: ‘It’s too much mentally … [the] mental aspect for me to take on what it means to be sustainably living … so if I have to go to McDonald’s … ’ (W2). Numerous whānau expressed disappointment and frustration about not being capable of sustainable and healthy practices for their families. Single parents and parents with infants or multiple children were notably confronting more challenges.

Perhaps because of the challenges, there was also great pride amongst participants for any sustainability successes and the hard work involved. The ‘hacks’ or ‘workarounds’ they had discovered were excitedly shared in Centre forums and discussions. Staff may have found it easier to negotiate challenges because their experiences were shared in the workplace.

Government and industry bodies in Aoteaoa NZ were widely felt by members of this community to be quite far behind in responding to shifting demand for sustainable food access. They also felt there may be more demand for sites like the Centre than are currently on offer, often referring to the Centre’s long waitlist as evidence.

## Discussion

Mutual learning and social change through community-based action and support were widely evident at the Centre as they reoriented to a more sustainable ethos of consumption. These show how ‘small changes in communities’ could help build wider social change (Swinburn et al. 2019). The Centre appears unique in its ability to operate outside of what is expected or mainstream for a small, community institution, however its success is partly due to motivated leaders leveraging their capacity to reorient established institutional systems to be increasingly sustainability focused (Swinburn et al. 2019).

Leadership was clearly vital for initiating and sustaining change, and for sharing both a vision of a ‘whole ethos’ and a culture of shared incremental learning. To motivate most people to risk becoming involved in the process of change, these leaders needed to present ideas that are high-quality, beneficial, well-timed and meaningful (Ramsden 2002). As the Centre developed and sustained operations to move away from more common food practices or social norms, they also systematised these ideas into their daily and formal practices, policies, and principles, and regularly reflected on and evaluated their efforts. The development of sustainability efforts and social support amongst staff and community was enhanced by the shared vision of change at the Centre (Graça et al. 2022). The ability of these leaders to consistently demonstrate their values and to recognise opportunities for building the capacities of others in the community helped in moving away from convenient and accepted norms and ideals about food, resources and waste (Balázs et al. 2016).

This research also shows how relationships matter in making this transition. Sharing interest and passion motivated participants, as did a shared culture of experimentation, acceptance of mistakes, understanding of waning and waxing enthusiasm, and of challenges (Crary et al. 2022). Sharing ‘hacks’ offered workarounds when larger, structural, or capability-inhibiting factors became challenging. These opportunities to share in learning and to be involved in the process enhanced willingness to connect and change among the Centre staff and community (Spadafore 2020), as did offering appealing and nutritious plant-based meals and ideas about alternative practices (Carvalho et al. 2022). Staff skill levels gradually increased, certainly enhanced by the expertise of the on-site chef (Crary et al. 2022; Graça et al. 2022). The relationships built through these practices further enhanced a sense of community and belonging to something valuable (Balázs et al. 2016). Framing the process positively and emphasising a culture of shared learning, experimentation, and fun, rather than focusing on loss or removal, also seemed to be key (Carvalho et al. 2022).

Our case study shows that a meaningful start in new practices can be an opportunity for long-term shifts if social support is sustained through ongoing sharing of practices (Spadafore 2020). This was particularly needed with the lack of institutional or larger, structural support (e.g. where government funding for a dedicated cook and food-planner is not easily available). The experiences of staff also speak to how motivations to change behaviours may require the structural support, capacity, and opportunities to match (Spadafore 2020; Graça et al. 2022). In the highly social setting of the Centre itself, the systematised aspects of sustainability might offer support and resources (like time and strategic planning) which whānau are much less likely to experience at home. Collectively, the ‘ripple effects’ of change were linked to relationships, connections and shared learning. What feels a great leap may actually be more of a pivot.

Challenges to engaging with sustainable consumption were sometimes internalised by individuals as guilt and overwhelm, and big shifts in practices were more difficult when people felt they were on their own or unsupported. A collective ethos or culture of ongoing, shared mutual learning, experimenting, and incremental but determined change, was key in the success of the transition. Reframing challenges as structural barriers to be overcome through collective efforts and learning rather than seeing them individual inadequacies to be ashamed of was also a helpful strategy (Carvalho et al. 2022).

### Limitations

This was a single-site case study and more research is vital to explore how helpful our findings could be to understand and develop sustainability in other community settings. We have endeavoured to provide enough context that these findings could be transferable to other examples.

## Conclusion

Food system change is urgently needed to improve health for people and planet. This case study provides insights into how change can happen at a localised community level, and how initiatives promoting food system change might be experienced by the people they are meant to involve or benefit, alongside what challenges are faced. This research highlights the essential role of leadership, shared meaning and vision, relationships. Mutual, incremental, and intentional learning and values integrated into everyday systems are also critical to achieving sustained transition. Our case study also highlights the value of framing challenges as external and tackling them in constructive and collective ways. Opportunities for mutual learning were carefully nurtured and developed and played a key role in the success of the Centre in transitioning to more sustaining ways of working.
